# CORRELATES OF SLEEP HEALTH AMONG OLDER PEOPLE WITH AND WITHOUT HIV IN UGANDA

**DOI:** 10.1093/geroni/igad104.3249

**Published:** 2023-12-21

**Authors:** Moka Yoo-Jeong, Aneeka Ratnayake, Brianne Olivieri-Mui, Yao Tong, Alexander C Tsai, Samson Okello, Stephen Asiimwe, Mark Siedner

**Affiliations:** Northeastern University, Boston, Massachusetts, United States; Northeastern University, Boston, Massachusetts, United States; Northeastern University, Boston, Massachusetts, United States; Harvard University, Cambridge, Massachusetts, United States; Harvard University, Cambridge, Massachusetts, United States; Harvard T.H. Chan School of Public Health, Boston, Massachusetts, United States; Massachusetts General Hospital, Boston, Massachusetts, United States; Harvard University, Cambridge, Massachusetts, United States

## Abstract

The proportion of older people in Uganda is expected to grow rapidly. Sleep issues disproportionately affect older people and people with HIV (PWH). Identifying determinants of sleep health is essential in the development of interventions. Thus, we assessed sleep-related factors in older Ugandans (aged ≥50 years) using cross-sectional data from the Quality of Life and Aging with HIV in Rural Uganda study. Sleep outcomes were measured by the Pittsburgh Sleep Quality Index (quality, duration, efficiency). The associations between sleep outcomes and potential determinants (loneliness, urban/rural setting, depression, anxiety, alcohol use, physical activity) were evaluated using multivariable linear regressions. Of 556 participants, 271 were PWH and 285 were people without HIV (PWoH). The two groups were similar in age, sex, and education. Depression screening via the 13-item Hopkins Symptoms Checklist was positive in 131 participants (PWH: 28.2%; PWoH: 21.6%; p=.05). There were no differences in sleep outcomes by HIV serostatus. Mean sleep quality was 0.89 (SD=0.78; range=0-3, 0=very good to 3=very bad), mean sleep duration was 6.5 hours (SD=1.7), and mean sleep efficiency was 74% (SD=19.5%; >85% is optimal). Depressive symptoms were associated with worse sleep quality (b=0.58; p<.001), reduced sleep duration (b=-0.94; p<.001), and worse sleep efficiency (b=-0.12; p<.001) regardless of HIV serostatus. Interestingly, older age was related to increased sleep duration (B=0.04, p=.001). Interventions targeting depression among older Ugandans may help with sleep. Longitudinal studies are needed to determine the bi-directionality of this relationship and further elicit potential pathways to support sleep health among older Ugandans.

